# 2017 update on the relationship between diabetes and colorectal cancer: epidemiology, potential molecular mechanisms and therapeutic implications

**DOI:** 10.18632/oncotarget.14472

**Published:** 2017-01-03

**Authors:** Nieves González, Isabel Prieto, Laura del Puerto-Nevado, Sergio Portal-Nuñez, Juan Antonio Ardura, Marta Corton, Beatriz Fernández-Fernández, Oscar Aguilera, Carmen Gomez-Guerrero, Sebastián Mas, Juan Antonio Moreno, Marta Ruiz-Ortega, Ana Belen Sanz, Maria Dolores Sanchez-Niño, Federico Rojo, Fernando Vivanco, Pedro Esbrit, Carmen Ayuso, Gloria Alvarez-Llamas, Jesús Egido, Jesús García-Foncillas, Alberto Ortiz, Diabetes Cancer Connect Consortium

**Affiliations:** ^1^ Renal, Vascular and Diabetes Research Laboratory, IIS-Fundacion Jimenez Diaz-UAM, Spanish Biomedical Research Network in Diabetes and Associated Metabolic Disorders (CIBERDEM), Madrid, Spain; ^2^ Radiation Oncology, Oncohealth Institute, IIS-Fundacion Jimenez Diaz-UAM, Madrid, Spain; ^3^ Translational Oncology Division, Oncohealth Institute, IIS-Fundacion Jimenez Diaz-UAM, Madrid, Spain; ^4^ Bone and Mineral Metabolism laboratory, IIS-Fundacion Jimenez Diaz-UAM, Madrid, Spain; ^5^ Genetics, IIS-Fundacion Jimenez Diaz-UAM, Madrid, Spain; ^6^ Nephrology, IIS-Fundacion Jimenez Diaz-UAM, Madrid, Spain; ^7^ REDINREN, Madrid, Spain; ^8^ Pathology, IIS-Fundacion Jimenez Diaz-UAM, Madrid, Spain; ^9^ Immunology, IIS-Fundacion Jimenez Diaz-UAM, Madrid, Spain; ^10^ Membership of the DiabetesCancerConnect Consortium is provided in the Acknowledgments

**Keywords:** hyperglycemia, inflammation, diabetic kidney disease, colon cancer, diabetes mellitus

## Abstract

Worldwide deaths from diabetes mellitus (DM) and colorectal cancer increased by 90% and 57%, respectively, over the past 20 years. The risk of colorectal cancer was estimated to be 27% higher in patients with type 2 DM than in non-diabetic controls. However, there are potential confounders, information from lower income countries is scarce, across the globe there is no correlation between DM prevalence and colorectal cancer incidence and the association has evolved over time, suggesting the impact of additional environmental factors. The clinical relevance of these associations depends on understanding the mechanism involved. Although evidence is limited, insulin use has been associated with increased and metformin with decreased incidence of colorectal cancer. In addition, colorectal cancer shares some cellular and molecular pathways with diabetes target organ damage, exemplified by diabetic kidney disease. These include epithelial cell injury, activation of inflammation and Wnt/β-catenin pathways and iron homeostasis defects, among others. Indeed, some drugs have undergone clinical trials for both cancer and diabetic kidney disease. Genome-wide association studies have identified diabetes-associated genes (e.g. *TCF7L2*) that may also contribute to colorectal cancer. We review the epidemiological evidence, potential pathophysiological mechanisms and therapeutic implications of the association between DM and colorectal cancer. Further studies should clarify the worldwide association between DM and colorectal cancer, strengthen the biological plausibility of a cause-and-effect relationship through characterization of the molecular pathways involved, search for specific molecular signatures of colorectal cancer under diabetic conditions, and eventually explore DM-specific strategies to prevent or treat colorectal cancer.

## BACKGROUND

**Figure 1:**
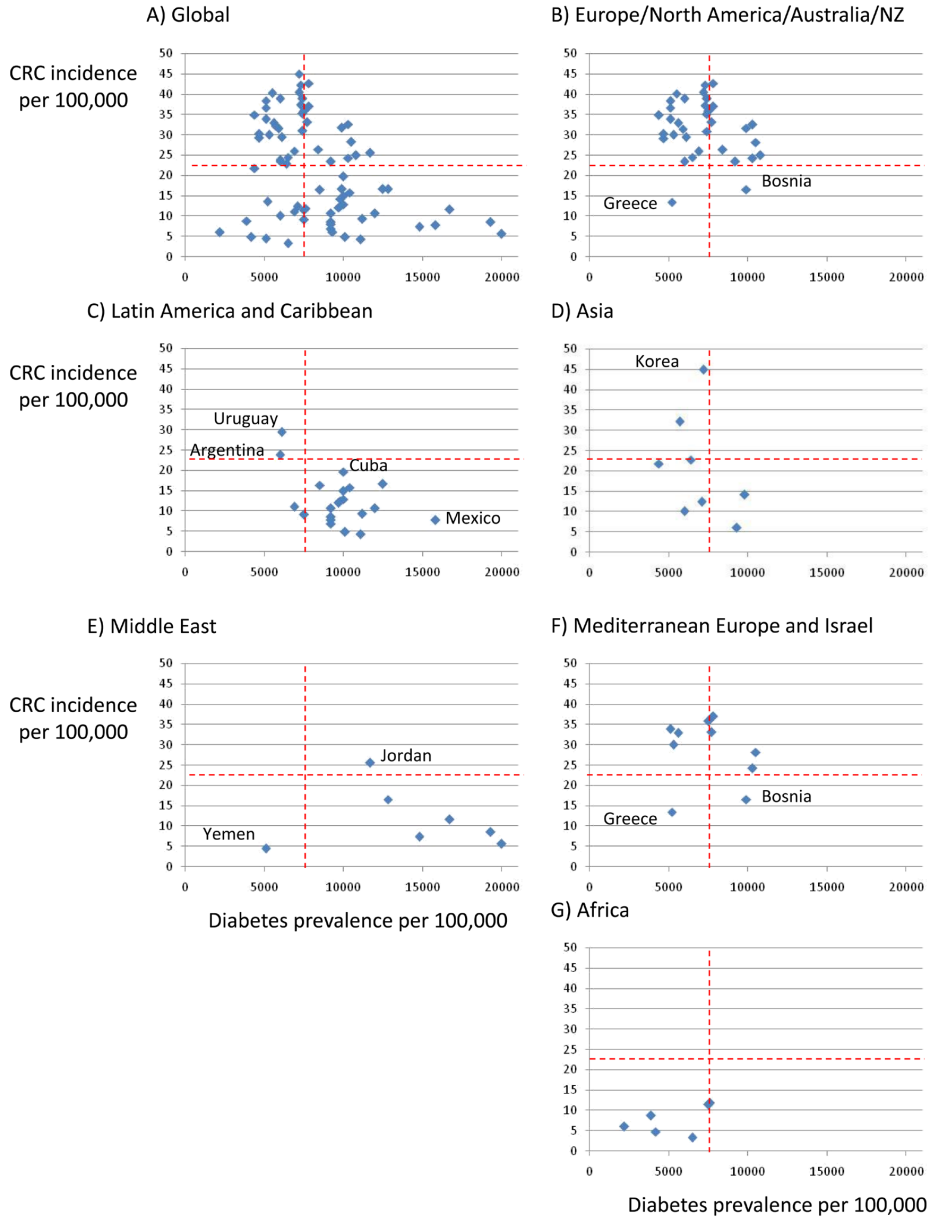
Relationship between incidence of colorectal cancer (CRC) and prevalence of DM in different parts of the world **A**. Global, **B**. Europe, North America and Australia/New Zealand, **C**. Latin America and Caribbean, **D**. Asia, **E**. Middle East, **F**. European Mediterranean countries and Israel, **G**. Africa. IDF 2015 data for DM (www.diabetesatlas.org/) and Globocan 2012 data for colorectal cancer (http://globocan.iarc.fr/Pages/age-specific_table_sel.aspx). Discontinuous red lines represent median values for the global population. Regional differences can be identified by the location of countries within the four quadrants. Note regional differences as well as countries that differ from others in the region. Regions are more clearly separated by CRC incidence than by DM prevalence, Europe/North America/Australia/NZ is the only high DM/high CRC region. Latin America and Caribbean is a high DM/low CRC region with the exception of Argentina and Uruguay where meat intake is high, while in the opposite extreme Mexico a is very high DM/low CRC country. In the Middle East a high prevalence of DM is not associated with high CRC incidence, unlike in European Mediterranean countries which in general behave as the rest of Europe. Korea is an example of low DM/high CRC country in Asia.

Diabetes mellitus (DM) and cancer are among the most frequent causes of death worldwide. According to Global Burden of Disease data, from 1990 to 2013 mortality from DM increased by 90% [1]. Colorectal cancer (CRC) is among the top causes of cancer death. From 1990 to 2013 global deaths from CRC increased by 57%, [1]. In the United States, CRC is the second leading cause of cancer death in men and women combined (http://www.ccalliance.org/colorectal_cancer/statistics.html) [2]. A link between DM and cancer is now recognized in American Diabetes Association (ADA) guidelines, following a 2010 consensus report [3, 4]. If the association holds, the current worldwide diabetes epidemic, fueled by life-style changes, may trigger a wave of CRC diagnoses. However, this knowledge has had limited impact on clinical care in the form of specific diagnostic tests or therapeutic approaches supported by clinical guidelines. Furthermore, on a worldwide basis the prevalence of DM and the incidence of CRC are not correlated, suggesting that country-specific factors may play a role in the association between DM and CRC (Figure 1). Annual CRC incidence rates vary more than ten-fold worldwide, the highest rates being in developed countries such as Korea (age-standardized rate 45 per 100, 000), Australia and Ireland, and the lowest in Western Africa (e.g. Cameroon 3.3 per 100, 000) (http://globocan.iarc.fr). By contrast, DM prevalence is highest in Egypt and United Arab Emirates (20, 000 per 100, 000, and lowest in Australia (5, 100), Ireland (4, 400) and Western Africa (www.diabetesatlas.org/). A better understanding of the factors underlying regional differences may provide clues to the relationship between DM and CRC. We now review the epidemiological evidence, potential pathophysiological mechanisms and therapeutic implications of the association between DM and CRC and propose a research agenda that may impact clinical practice to prevent or treat CRC in DM patients. A Pubmed search with the key words “(diabetes OR insulin OR hyperglycemia) AND (colon OR colorectal) AND cancer” was performed with no time cut-off points and further references added from the reference list of the publications found or based on the authors own experience knowledge.

## DIABETES MELLITUS

DM is characterized by hyperglycemia resulting from defects in insulin secretion and/or insulin action. Chronic hyperglycemia is associated with injury to the kidneys, heart, nerves, eyes and blood vessels [5]. In type 1 DM (T1DM, 5-10% of DM cases), cell-mediated autoimmune destruction of pancreatic β-cells causes absolute insulin deficiency. Type 2 DM (T2DM) is characterized by insulin resistance and relative insulin deficiency. T2DM patients are frequently obese and older at DM onset than T1DM patients [6]. Obesity promotes insulin-resistance and is thought to be a major driver of the current DM epidemic. Mendelian-inherited genetic defects of β-cells or of the insulin signaling machinery also cause DM [5].

Mean age at diagnosis of DM is 54 years in the US (http://www.cdc.gov/diabetes/statistics/age/). Therapies for DM increase insulin availability (insulin or insulin analog administration or agents that promote insulin secretion), improve sens itivity to insulin, decrease glucose synthesis, delay the gut absorption of carbohydrate, or increase urinary glucose excretion (Supplementary Table 1). The preferred initial and most widely used pharmacological agent for T2DM is metformin, which decreases glucose production by inhibiting the mitochondrial glycerophosphate dehydrogenase (GPDH, GPD2) [7]. If adequate glucose control is not achieved within 3-6 months, a second oral agent, a Glucagon-like peptide-1 (GLP-1) receptor agonist, or insulin should be added [8, 9].

## COLORECTAL CANCER

CRC originates from colon epithelium [10]. Over 70% CRCs are sporadic, resulting from dietary and environmental factors. The incidence increases with age and they usually occur over the age of 50 years. True inheritable CRC (<10% of cases) may be associated or not to colonic polyps (Table 1) [11]. The familial type (25% of cases) is associated with a family history of CRC or large adenomas, in the absence of classic Mendelian inheritance [12]. Right- and left-sided CRCs exhibit different epidemiological patterns, sensitivities to chemotherapy and outcomes, probably related to different molecular characteristics and chromosomal instability with left-sided tumors [13].

**Table 1:** Genetics of colorectal cancer and potential impact of DM on colorectal cancer-related genes

Colorectal cancer	Mutation	Inheritance	Impact of DM on gene expression *	Reference
Familial adenomatous polyposis	Inactivating germline mutation in adenomatous polyposis coli (*APC*)	Autosomal dominant	Increased *APC*	[283,284]
MUTYH-associated polyposis	Inactivating germline mutation in *MUTYH*	Autosomal recessive	Unchanged *MUTYH*	[283,284]
Peutz-Jeghers syndrome	Inactivating germline mutation in serine threonine kinase 11 (*STK11*)	Autosomal dominant	Increased *STK11*	[285]
Hereditary non-polyposis colorectal cancer (Lynch syndrome)	Inactivating germline mutation in *MLH1, MSH2, MSH6*, or *PMS2*	Autosomal dominant	Unchanged *MLH1, PMS2* Increased *MSH2, MSH6*	[286]
Chromosomal instability (frequent)	Acquired accumulation of numerical (aneuploidy) or structural chromosomal abnormalities and mutations in specific oncogenes and tumor suppressor genes (e.g. *APC, PIK3CA, SMAD4, KRAS, TP53, BRAF*)		Unchanged *PIK3CA, SMAD4, BRAF* Increased *KRAS, TP53*	[287–289]

* Kidney gene expression in human diabetic kidney disease transcriptomics (http://www.nephromine.org).

CRC is initiated by mutations in tumor suppressor genes (adenomatous polyposis coli or *APC*, *CTNNB1, p53*) and oncogenes (*KRAS*). Accumulation of multiple mutations leads to a selective growth advantage for transformed epithelial cells that is modulated by epigenetic changes [14, 15]. Diet, the microbiota and the inflammatory response to the microbiota are potential players [16–22]. Indeed, chronic gut inflammation (e.g. ulcerative colitis or Crohn´s disease) is associated with increased incidence of colon cancer. A major molecular pathway is Wnt signaling activation of the transcription factor β-catenin to promote expression of cell proliferation genes. Loss-of-function mutations or epigenetic silencing of *APC* leads to aberrant β-catenin accumulation and uncontrolled cell proliferation. The normal APC protein forms a complex with glycogen synthase kinase 3-beta (GSK-3β) that allows GSK-3β to phosphorylate β-catenin, targeting it for ubiquitination and proteasomal degradation, thus decreasing β-catenin-dependent transcriptional events [23].

Early-stage CRC is treated with surgery and locally advanced CRC (radically resected stage III and ‘high-risk’ stage II disease) with adjuvant chemotherapy on top of surgery. Rectal cancer with nodal disease standard treatment includes neoadjuvant chemo-radiation [24]. Adjuvant chemotherapy schemes contain 5-fluorouracil and oxaliplatin. Metastatic CRC is treated with irinotecan or oxaliplatin combined with a fluoropyrimidine and leucovorin (FOLFIRI or FOLFOX regimens) [25]. Addition of targeted therapies over the past 10 years has improved overall survival. Testing for KRAS, NRAS, BRAF, PIK3CA and PTEN mutations is used to assess the potential clinical benefit of anti-Epidermal Growth Factor Receptor (anti-EGFR) and panitumumab treatment. Meta-analyses suggest that mutation testing for KRAS exon 2 is the strongest biomarker of response. The addition of anti-Vascular Endothelial Growth Factor (anti-VEGF) agents (bevacizumab, regorafenib) to chemotherapy of metastatic CRC prolongs progression-free and overall survival in first- and second line therapy [26].

## EPIDEMIOLOGICAL ASSOCIATION BETWEEN DIABETES AND CRC

Epidemiological studies suggest that DM, especially T2DM, is associated with increased risk of cancer at several sites, including CRC [27] (Table 3). The first prospective association was reported in 1998 in US participants followed from 1960 to 1972 [28]. The adjusted incidence density ratio of CRC was 1.30 (95% confidence interval (CI) 1.03-1.65) for diabetic males, but not significant for females. The association was found only among non-smoker males. A more recent prospective US study followed an older cohort from 1995 to 2004 and observed an increased adjusted Hazard Ratio (HR) for CRC in both males and females [29]. Lifestyle changes from the 60s to the 90s may explain the change in female risk. A similar association has been reported in Japan [30], China [31], Australia [32] or certain European countries (e.g. Sweden) [33], among others. A recent umbrella review of meta-analyses of observational studies on T2DM and cancer updated to the end of 2013 concluded that CRC was one of only four cancer sites associated to T2DM with robust supporting evidence and without hints of bias [34]. Furthermore, in a meta-analysis of prospective cohort studies encompassing near a million participants, prediabetes (impaired fasting glucose and/or impaired glucose tolerance) was also associated with increased risk of CRC [35]. However, uncertainties remain. The presence of detection bias and/or reverse causation has been suggested by studies in Australia, Israel and the Netherlands that found a higher risk of cancer within 3 months of a DM diagnosis [32, 36, 37]. In this regard, in the US, respondents with diabetes were 22% more likely to be up-to-date on CRC screening than those without diabetes [38]. A higher risk of developing DM within 5 years of CRC diagnosis was also reported [39]. In addition, regional differences exist: in Norway and the Netherlands only diabetic females had a higher incidence of proximal colon cancer or CRC [40, 41], while no association was found in Tyrol. Unraveling the reasons underlying regional differences may provide clues to the association and to public health interventions. Potential differences in the use of specific antidiabetic drugs may play a role as discussed below. Furthermore, epidemiological data from developing countries are scarce. This is an important piece of missing information since almost 55% of CRC cases occur in more developed regions (http://globocan.iarc.fr/Pages/fact_sheets_cancer.aspx), while 80% of DM patients live in low- and middle-income countries (www.diabetesatlas.org/).

**Table 3:** Epidemiological association between DM and risk of CRC

Country	N (x1000)	Mean age (years)	Period (years)	Location	Males	Females	Overall	Ref
US*	850	54	59-72	CRC	1.30 (1.03-1.65)	1.16 (0.87-1.53)	Not available	[28]
US**	484	62	95-06	CRC	Colon 1.24 (1.12-1.38) Rectum 1.34 (1.14-1.57)	Colon 1.37 (1.16-1.60) Rectum 1.43 (1.08-1.88)	Colon 1.27 (1.17-1.39) Rectum 1.36 (1.18-1.56)	[29]
Japan***	335	N.A.	N.A.	CRC	N.A.	N.A.	1.40 (1.19-1.64)	[30]
China****	327	60	07-13	CRC	Colon 1.47 (1.29-1.67) Rectum 1.25 (1.09-1.43)	Colon 1.33 (1.15-1.54) Rectum 1.29 (1.10-1.51)	Colon 1.40 (1.27-1.55) Rectum1.26 (1.14-1.40)	[31]
Australia****	953	27 (T1DN) 60 (T2DN)	97-08	CRC	1.18 (1.15-1.21)	1.16 (1.13-1.20)	N.A.	[32]
Sweden****	2.9 1.4	N.A.	64-10	CRC	N.A.	N.A.	Colon 1.33 (1.28-1.38) Rectum 1.19 (1.13-1.25)	[33]
Norway***	751 pers/year	71	84-96	CRC	CRC 0.66 (0.35-1.34)	CRC 1.55 (1.04-2.31) Colon 1.60 (1.02-2.51) Rectum 2.70 (1.29-5.61)	N.A.	[40]
Tyrol****	5.7	58	88-10	CRC	1.11 (0.81-1.49)	0.94 (0.62-1.36)	N.A.	[290]
Israel**	2186	64	02-12	CRC	1.45 (1.37-1.55)	1.48 (1.39-1.57)	N.A.	[36]
Netherlands**	120	62	86-06	CRC	CRC 0.95 (0.75–1.20) Proximal 1.13 (0.76-1.68) Distal 0.77 (0.49–1.21) Rectum 0.50 (0.21–1.22)	CRC 1.08 (0.85–1.37) Proximal 1.44 (1.05–1.99) Distal 0.75 (0.44–1.27) Rectum 1.16 (0.54–2.48)	N.A.	[41]
Meta-analysis***	8244	N.A.	N.A.	CRC	N.A.	N.A.	1.27 (1.21-1.34)	[34]

95% confidence interval shown. * Adjusted incidence density ratio: ** Adjusted HR; ***RR. **** Standardized incidence ratios

N.A.: not available

Risk factors shared by CRC, DM, and DM target organ damage may be confounders in epidemiological studies (Table 2). Obesity is a major risk factor for T2DM, cancer and diabetic kidney disease (DKD) [42, 43]. However, key studies observing an association between DM and CRC were adjusted by BMI. In this regard, there may be a relationship between obesity, insulin resistance and CRC. In a prospective European study, lower CRC risk was observed for metabolically healthy/overweight individuals compared with metabolically unhealthy/overweight individuals, defined as individuals with higher C-peptide levels indicative of hyperinsulinaemia [44]. Diet may be another confounder. A high meat intake increases and a Mediterranean diet decreases both the risk of DM and of CRC [45].

**Table 2:** Key risk factors for T2DM, colorectal cancer and DM complications (Diabetic kidney disease)

Risk factor	T2DM	Colorectal cancer	Diabetic kidney disease
**Race**	African American, Native American	African American	African American, Native American
**Obesity**	Yes	Yes	Yes
**Inflammation**	Yes	Yes	Yes
**Microbiota**	Yes	Yes	Unknown
**Low vitamin D**	Yes	Yes	Yes
**High protein (meat protein) diet**	Yes	Yes	Yes
**Low fiber diet**	Yes	Yes	ND
**No Mediterranean diet**	Yes	Yes	ND
**Low magnesium intake/hypomagnesemia**	Yes	Yes	Yes
**Angiotensin II**	Yes	Yes	Yes
**Age**	Yes	Yes	Unclear

## POTENTIAL MOLECULAR MECHANISMS OF THE ASSOCIATION BETWEEN DM AND CRC

**Figure 2:**
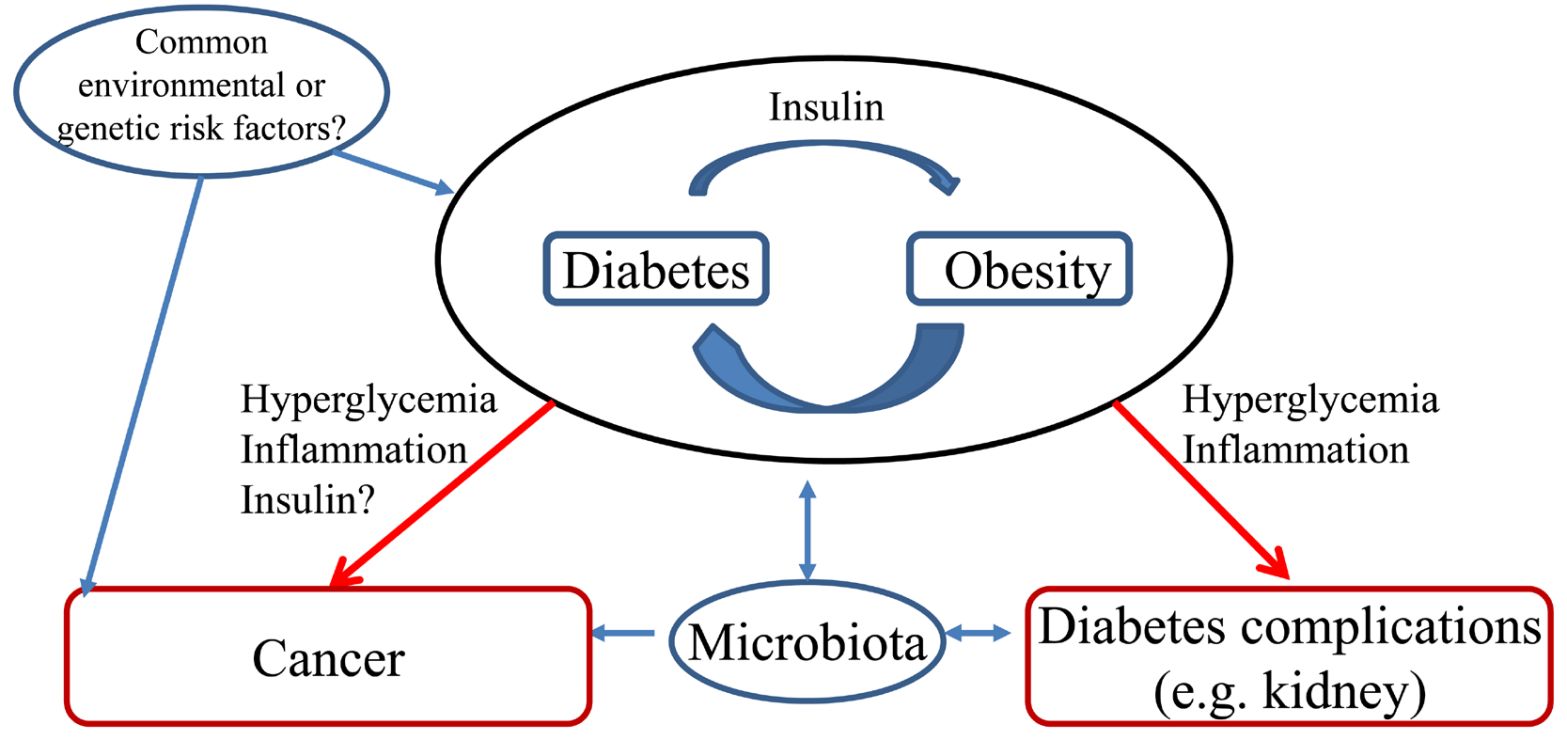
Hypotheses potentially explaining the association between diabetes and colorectal cancer Two major potential relationships have been depicted. **A**. Common risk factors (e.g. diet, genetic) favor both diabetes and colorectal cancer; **B**. Diabetes favors cancer development. These potential relationships are put in context with the occurrence of other diabetes complications such a chronic kidney disease. Obesity is a known risk factor for both colorectal cancer and diabetic kidney disease.

The association between DM and CRC may result from shared risk factors between T2DM and cancer but epidemiological data suggest a potential contribution of hyperinsulinemia, hyperglycemia or DM therapy [4, 46, 47] (Figure 2). Additionally, the DM microenvironment, such as advanced glycation end-products (AGEs), hyperlipidemia, local inflammation/oxidative stress, extracellular matrix alterations, and altered microbiota or ischemia due to vasculopathy may recruit secondary mediators of injury that may favor the development of both cancer and other complications of DM such as DKD.

### Insulin

Insulin and insulin-like growth factor (IGF)-1 have growth factor and antiapoptotic properties in a variety of cultured tumor and non-tumor cell types, including normal colon epithelium and colon cancer cells [48, 49]. These actions have been interpreted as part of a putative tumor-enhancing effect of insulin [50, 51]. However, insulin signaling is also required for survival and function of healthy cells in vivo, such as podocytes, key cells in DKD, and selective podocyte insulin resistance reproduces features of DKD in the absence of hyperglycemia [52]. The mTOR and p21-activated protein kinase-1 (PAK-1)/Wnt/β-catenin intracellular pathways are involved in insulin-stimulated proto-oncogene expression in intestinal cells [53]. These molecular pathways also mediate diabetic complications, including DKD [54].

An increased incidence of azoxymethane-induced intestinal tract cancer was observed in preclinical models of obesity and T2DM, including obese Zucker rats and KK Ay, db/db and ob/ob mice [55–57]. The addition, the incidence and multiplicity of intestinal adenomas was higher in db/db mice with Apc mutations than in non-diabetic mice [58]. However, the relative role of hyperglycemia, hyperinsulinemia or obesity was not characterized.

The role of hyperinsulinemia was studied in a normoglycemic model of mammary cancer growth, but results do not necessarily extrapolate to CRC [59]. A tyrosine kinase inhibitor specific to the insulin and IGF-1 receptors aggravated hyperinsulinemia but prevented insulin signaling and cancer growth. However, tyrosine kinase inhibitors are promiscuous and are in clinical use as anti-tumor agents. Thus, the fact that members of an anti-tumor agent family decrease tumor growth is not definitive evidence for a role of insulin. CL-316243, a β3-adrenergic receptor agonist that sensitizes to insulin action, reduced hyperinsulinemia and phosphorylation of insulin and IGF-1 receptors and attenuated mammary tumor progression, supporting a role for hyperinsulinemia in T2DM associated tumor progression [60].

### Hyperglycemia

Hyperglycemia has been implicated both in colon cancer growth and in DKD and some of the molecular mechanisms are shared by both diseases. High glucose levels and AGEs increase proliferation and migration of cultured colon cancer cells [61, 62]. High glucose levels also enhance resistance to 5-fluoruracil-induced apoptosis [63]. AGE-induced CRC cell proliferation requires carbohydrate response element-binding protein (ChREBP) [64], a key transcription factor also involved in DKD [65]. The polyol and hexosamine pathways, which increase glucose oxidation, are upregulated in diabetes target organ epithelial cells [54] and in colon cancer [66]. Hyperglycemia and AGEs induce oxidative stress and inflammation, which can damage cellular components and contribute to malignant cell transformation [67–69]. High glucose-induced oxidative stress plays a pivotal role in the development of diabetes complications by activating different pathways, such as the transcription factor nuclear factor-kappa B (NF-κB) [70, 71]. Indeed, bardoxolone methyl, a potent nuclear factor erythroid 2-related factor 2 (Nrf2) activator/NF-κB inhibitor, improved glomerular filtration in RCT in DKD [72]. Interestingly, the observation that bardoxolone increased glomerular filtration was first made in clinical trials exploring its anticancer potential.

The Warburg effect refers to the high glucose uptake and metabolism of glucose through glycolysis rather than aerobic phosphorylation in tumor cells despite the presence of oxygen [73, 74]. Glycolysis is less efficient but generates adenosine triphosphate (ATP) faster, conferring a growth advantage to tumor cells. Upregulation of insulin-independent glucose transporters such as glucotransporter-1 (Glut-1) favors glucose uptake by cancer cells [75, 76]. Glut overexpression is usually translated into higher proliferation rates. The diabetic milieu and transforming growth factor (TGF)-β1 upregulate renal cell Glut-1 and this is thought to contribute to the pathogenesis of DKD [77].

Few preclinical studies have addressed the impact of hyperglycemia *per se* (i.e. T1DM) on colon cancer. Streptozotocin-induced hyperglycemia, an insulin-deficiency DM model, increased liver metastasis of mouse colon cancer cells, while glycemic control with either insulin or gliclazide was protective [78]. These studies suggest that hyperglycemia per se may favor colorectal tumor growth and that hyperglycemia may be a more powerful stimulus for tumorigenesis than insulin in experimental animals.

**Figure 3:**
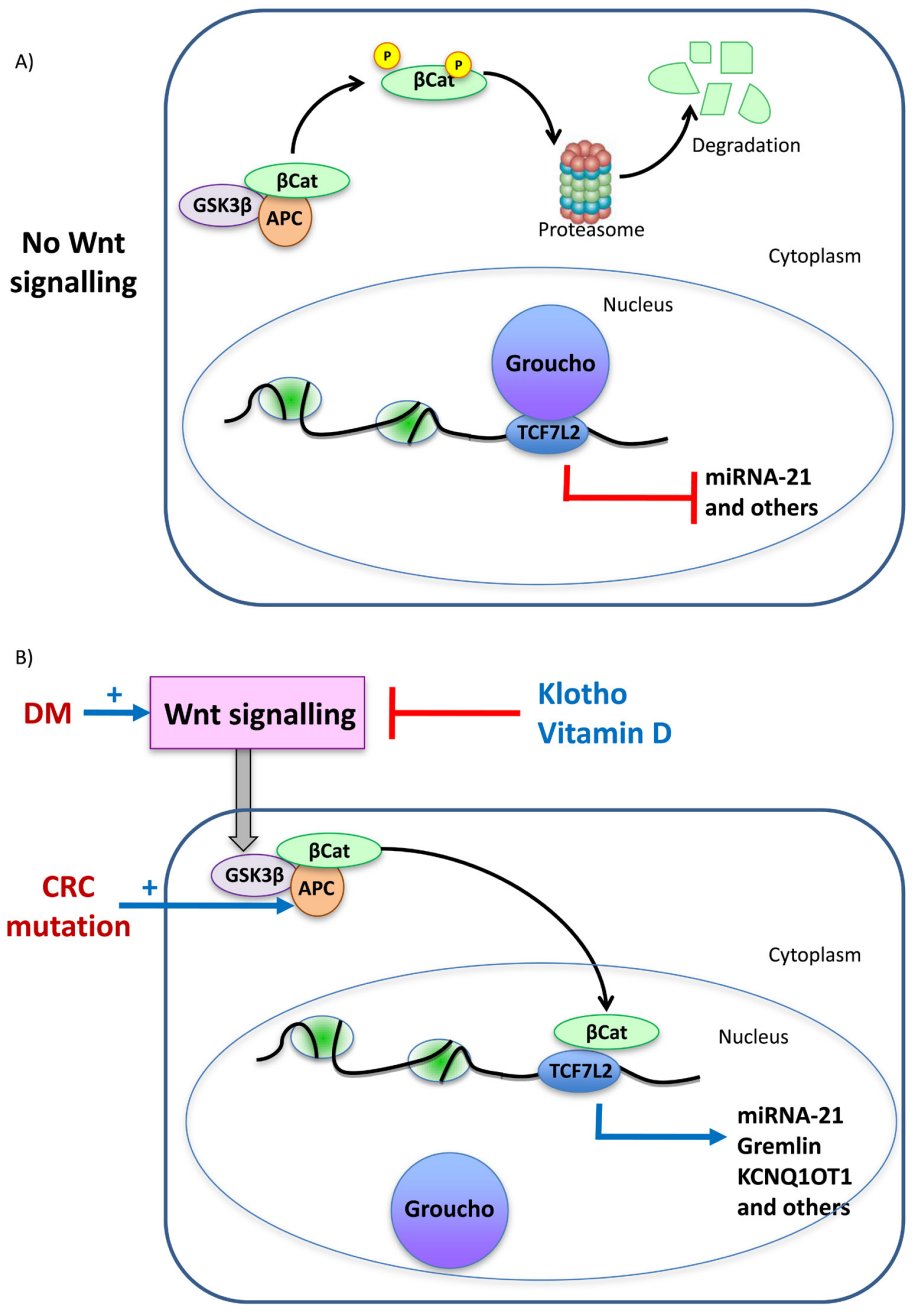
Key molecular pathways potentially linking diabetes and colorectal cancer The example of β-catenin activation. **A**. In the absence of Wnt signaling, APC-bound glycogen synthase kinase 3-beta (GSK-3β) phosphorylates β-catenin (βCat), targeting it for ubiquitination and proteasomal degradation. In the absence of nuclear β-catenin, Groucho binds to transcription factors of the TCF family, repressing transcription. The TCF family includes TCF7L2 which has been associated to DM, DM complications and colon cancer by GWAS studies. **B**. Colon cancer is characterized by loss of function mutations of APC and in DM Wnt signaling is activated. Klotho and vitamin D prevent Wnt signaling and are protective against tumors and against DM complications. Wnt signaling prevents β-catenin phosphorylation and degradation allowing its nuclear migration, where it displaces Groucho and promotes transcription of genes involved in cell proliferation as well as other genes such as miR-21. miR21 contributes to tumorigenesis and to diabetes complications such as kidney injury. GWAS identified a *GREM1* SNP associated with CRC susceptibility that facilitates TCF7L2 binding to DNA, leading to stronger *GREM1* gene expression. A *GREM1* SNP also associate with diabetic kidney disease. The gene product, Gremlin, promotes kidney injury in DM as well as colon cancer cell migration. *KCNQ1* was associated with T2DM by GWAS. This locus encodes KCNQ1OT1, a β-catenin target upregulated in CRC.

Wnt/β-catenin is activated in CRC as a direct consequence of *APC* mutations and in kidney cells in DKD [79], protecting glomerular mesangial cells from high-glucose-mediated cell apoptosis [80] but causing podocyte dysfunction and proteinuria [79]. β-catenin expression and altered phosphorylation, and cell proliferation were higher in normal colon epithelium surrounding tumor tissue in diabetic than in non-diabetic patients [81]. VDR activation antagonizes Wnt/β-catenin signaling [82] (Figure 3). The nephroprotective action of VDR activators has been related to Wnt/β-catenin inhibition [83]. Vitamin D deficiency is common in DM [84] and has also been associated with increased risk of CRC [85, 86]. High-glucose-induced inflammatory and fibrogenic responses in kidney cells contribute to DKD and are prevented by vitamin D receptor (VDR) activation [87–90]

EGFR signaling contributes to tumorigenesis and tumor progression of CRC and EGFR-targeted cetuximab is used to treat CRC. Genetic or pharmacological EGFR blockade slows experimental renal disease progression [91]. High-glucose, AGE, angiotensin II, and pro-inflammatory cytokines, such as TWEAK and parathyroid hormone-related protein (PTHrP) AGE promote EGFR transactivation in kidney cells [92–96]In this regard, TWEAK targeting antibodies are undergoing clinical trials in kidney disease, while targeting the TWEAK receptor Fn14 reduced colon cancer metastasis in experimental animals [95, 97]. Inhibition of EGFR with erlotinib attenuates DKD in experimental T1DM, through inhibition of mTOR [98]. Indeed, mTOR is activated in diabetic podocytes and mTOR targeting protects from DKD [99]. CCN2 is a novel EGFR ligand that promotes kidney inflammation and DKD progression [100, 101] and in CRC cells, regulates cell migration and prevents apoptosis [102].

Klotho is an anti-aging hormone of kidney origin with anti-inflammatory and anti-fibrotic properties [103, 104]. Experimental and human diabetes, inflammation and hyperlipidemia are associated with decreased Klotho expression [105–108]. Loss of Klotho contributes to kidney injury by de-repression of Wnt/β-catenin signaling [109] and similar mechanisms may be active in colon cancer cells. In this regard, Klotho suppresses growth and invasion of colon cancer cells through inhibition of the IGF1R-mediated PI3K/Akt pathway [110] and is frequently inactivated through promoter hypermethylation in CRC [111].

### Inflammation and microbiota

Inflammation is a critical component of diabetes-induced target organ injury and of CRC initiation and progression [112, 113]. In preclinical models of T2DM, inflammation contributed to carcinogenesis and tumor growth, which were prevented by TNF-neutralizing monoclonal antibodies [57].

Multiple signaling pathways are involved in the inflammatory response, including MAPK, NF-κB, janus kinase/signal transducer and activator of transcription (JAK/STAT) and hypoxia-inducible factor-1α [68, 114–117]. Persistent NF-κB/IL-6/STAT3 activation promotes colitis associated CRC [118]. The non-canonical NF-κB pathway has also been implicated in diabetes complications and cancer [119–121]. The upstream kinase of this pathway, NIK, contributes to β cell failure in diet-induced obesity [122], promotes kidney injury [123] and underlies the sensitivity of Nlrp12^−/−^ mice to gut inflammation and tumorigenesis [124]. These intracellular pathways amplify inflammatory responses and promote angiogenesis, cancer growth and invasiveness of malignant cells [125, 126], as well as progression of diabetes target organ injury such as DKD [117].

The interaction between colon epithelial cells and the microbiota may confer susceptibility to both colon cancer and obesity. The inflammasome regulates the microbiota and the inflammatory response of epithelial cells to the microbiota. Deficiency in inflammasome components (e.g. Nlrp6) is associated with an abnormal microbiota, exacerbated gut inflammatory responses [127] and colon tumorigenesis [128] dependent on microbiota-induced activation of epithelial IL-6 signaling [17]. Microbiota-dependent inflammatory responses may contribute to non-Mendelian familial aggregation of colon cancer since in preclinical models the risk of cancer was transmissible between co-housed individuals with the microbiota. The gut microbiota also impacts host metabolism, facilitating obesity, insulin resistance and T2DM [129]. Thus, inflammasome deficiency-related changes in gut microbiota are associated with insulin resistance and obesity [130]. In this regard, T2DM is one of three models of microbiome-associated human conditions to be studied by the Integrative Human Microbiome Project (iHMP, http://hmp2.org) [131]. Human T2DM and CRC share some microbiota features, such a decrease in the abundance of butyrate-producing bacteria [18, 132]. Butyrate is a breakdown product of dietary fiber that has anti-tumorigenic properties and is associated with decreased incidence of CRC [18]. In mice, the microbiota potential for butyrate production negatively correlated with tumor count [133]. Butyrate also has nephroprotective properties in DKD [134].

Iron metabolism. Altered iron metabolism facilitates rapid proliferation in cancer cells [135]. Indeed, constitutive Wnt/β-catenin signaling in colon cancer cells is iron-dependent [136] and iron chelation limits cell proliferation and has anti-inflammatory effects through NF-κB blockade [137]. Iron overload causes DM and is present in target organs of diabetes, such as the kidneys, while iron depletion upregulates glucose uptake and insulin signaling in liver and decreases kidney inflammation in experimental diabetes [138–140]. Indeed, the Trial to Assess Chelation Therapy (TACT) disclosed a benefit of ethylenediaminetetraacetic acid (EDTA), a chelator that also binds iron, on cardiovascular outcomes, especially in DM patients [141]. Thus, excess cellular iron may facilitate CRC growth, DM and DM complications. Heme iron may be the common denominator in the association of red meat intake with both DM and CRC [142, 143].

### Epigenetic changes

CRC and DM also share some epigenetic changes. Thus, both CRC and DM were associated with a positive septin 9 (SEPT9) DNA-methylation assay (Epi-proColon) result [144]. In this regard, SEPT9 is differentially methylated in human T2DM islet cells and was shown to perturb insulin and glucagon secretion [145].

miRNAs are small non-coding RNA molecules that regulate gene expression. Pathogenic miRNAs may be shared by CRC and DKD [146–148]. In murine DKD, renal miR-21 expression was increased and miR-21 knockdown ameliorated renal damage [149]. The pathogenic potential of miR-21 is supported by some, but not all additional reports [150, 151]. miR-21 is also part of a six-miRNA-based classifier that reliably predicts CRC recurrence [148, 152]. Functional studies support a role for miR-21 in colon cancer proliferation and invasion [153, 154] and targeting miR-21 enhanced the sensitivity of human colon cancer cells to chemoradiotherapy and reduced angiogenesis [154, 155]. Metformin synergy with 5-fluorouracil and oxaliplatin to induce death of chemoresistant colon cancer cells was also associated with a reduction in miR-21 [156].

## ADDITIONAL INFORMATION FROM SYSTEMS BIOLOGY APPROACHES

Genome-wide association studies (GWAS) have identified susceptibility genes for DM or CRC that provide insights into potentially shared pathogenic pathways, such as *TCF7L2, KCNQ1, HMGA2, RHPN2* and *GREM1*.

*TCF7L2* harbors common genetic variants with the strongest effect on T2DM risk [157–159] and on DM complications such as DKD [160] and is also susceptibility locus for CRC loci in East Asians [161]. TCF7L2 is a transcription factor and β-catenin transcriptional partner in the Wnt-signaling pathway. DNA-bound TCFs repress gene transcription in the absence of β-catenin, but are required for β-catenin transcriptional activity [162]. TCF7L2 also promotes miR-21 expression [163]. Another CRC-associated Single Nucleotide Polymorphism (SNP), rs6983267, is located at a TCF7L2 binding site and the risk allele results in stronger TCF7L2 binding, facilitating Wnt signaling [164]. A common *GREM1* SNP, rs16969681, associated with CRC susceptibility facilitates TCF7L2 binding to DNA leading to stronger gene expression [165]. A germline duplication upstream of *GREM1* causes hereditary mixed polyposis syndrome and Mendelian-dominant predisposition to CRC through ectopic *GREM1* overexpression in the intestinal epithelium [166, 167]. *GREM1* was initially identified as one of the most upregulated genes in cultured mesangial cells exposed to high glucose [168] and *GREM1* gene variants also associate with DKD [169]. Gremlin, the protein codified by *GREM1*, has been proposed as a key mediator of DKD [170–173]Gremlin promotes the motility of CRC cells [174] and the epithelial to mesenchymal transition in kidney tubular cells, also associated with increased motility [175, 176]. The precise role of TCF7L2 in CRC should be further defined. Thus, *TCF7L2* mutations identified in cancer samples abolish its ability to function as a transcriptional regulator and result in increased CRC cell growth [177]. Given the multitude of target genes, this is not surprising.

*KCNQ1* was associated with T2DM [178]. This locus encodes both KCNQ1 and the long noncoding RNAs (lncRNAs) *KCNQ1OT1*, which is a β-catenin target dysregulated in CRC [179]. In human CRC, low *KCNQ1* expression was associated with poor survival and mutation of the murine homologue Kcnq1 increased the risk for intestinal tumors [180].

*HMGA2* is a further gene associated to risk of T2DM and DKD in GWAS [158, 181]. *HMGA2* expression is increased in and promotes the malignant behavior of CRC [182, 183]. Conversely, CRC GWAS identified *RHPN2* as a susceptibility loci and *RHPN2* expression is upregulated in experimental DKD [184, 185].

Pathway-based enrichment analysis of 23 independent gene expression profiling studies on prognosis of CRC observed overrepresentation of the oxidative phosphorylation chain, the extracellular matrix receptor interaction category, and a general category related to cell proliferation and apoptosis [186]. These categories are functionally related with cancer progression. Eight of the genes were also present in a previous meta-analysis of ten expression profiling studies of differentially expressed genes in CRC with good versus bad prognosis, including *IQGAP1, YWHAH* and *TP53*. [186]. IQGAP1 is part of the podocyte filter for proteins and regulates the occurrence of proteinuria, the hallmark of DKD [187] and *YWHAH* expression was increased in human DKD transcriptomics studies [188, 189]. Furthermore, human DKD transcriptomics revealed that 25% of apoptosis-related genes were differentially regulated in kidney tissue [190–192]. Some of the specific factors identified by human DKD transcriptomics and functionally characterized to contribute to kidney injury, also promote CRC growth, such as the MIF/CD74 system which is under study as a therapeutic target in colon cancer [193, 194]. Furthermore, elements of the JAK/STAT, VEGFR signaling and inflammation-related pathways were also overrepresented in human DKD [184, 189]. JAK/STAT, VEGF and inflammation are therapeutic targets in cancer.

As part of the Human Proteome Project, the Biology/Disease-driven Human Proteome Project (B/D-HPP) consortium leads specific projects on diabetes (HDPP) and cancer that may shed some additional light on the relationship between both diseases [195]. Protein candidate markers responding to CRC existence (diagnosis), stratification (different response related to stage) or prognosis (survival/metastasis) have been identified [196–199]. Most studies compared normal (healthy) tissue with tumor. The top four regulated proteins in a systematic review of CRC were 60-kDa heat shock protein (HSP60) and Nucleoside diphosphate kinase A (nm23-H1), up-regulated, and Selenium-binding protein 1 (SELENBP1) and Carbonic anhydrase I (CAI), down-regulated [200]. Interestingly, expression of the HSPD1 gene encoding HSP60 was upregulated and SELENBP1 downregulated in human DKD, according to the Nephromine database, further suggesting potential common pathogenic pathways between DKD and colon cancer (http://www.nephromine.org/).

Bioinformatics approaches may be used to integrate the growing systems biology databases. One such approach, the Drug-specific Signaling Pathway Network (DSPathNet) was used to tentatively identify seven genes (CDKN1A, ESR1, MAX, MYC, PPARGC1A, SP1, and STK11) and one novel MYC-centered pathway that might play a role in metformin antidiabetic and anticancer effects [201]. Interestingly, PPARGC1A protects from kidney injury and the expression is downregulated by inflammation [202].

## IMPLICATIONS FOR THERAPY

Given the high and increasing incidence and prevalence of DM and CRC, it is likely that, independently from any common pathogenic pathways or associations, many DM patients will develop CRC. This brings the question whether physicians need to modify the approach to therapy of DM or CRC in diabetic patients with both conditions. In this regard, a diagnosis of cancer is frequently associated to a subsequent decrease in adherence to antidiabetic medication [203].

### Choice of antidiabetic agent in the patient with CRC

The ADA Standards of Medical Care in Diabetes indicates that patients with DM should be encouraged to undergo recommended age- and sex-appropriate cancer screenings and to reduce their modifiable cancer risk factors (obesity, smoking, physical inactivity) [9]. In the presence of cancer, higher HbA1c goals should be considered: <8% in the absence of metastases and <8.5% for patients with metastatic cancer. If indeed hyperglycemia underlies the higher incidence of colon cancer in DM, these higher thresholds may theoretically impair cancer-related outcomes.

ADA 2016 does not provide recommendations on the choice of antidiabetic treatment in patients with cancer or CRC [204]. However, the initial antidiabetic agent recommended for standard T2DM patients, metformin, has been associated with decreased incidence or better outcomes in cancer patients [205–207]. Thus, even if prospective clinical studies confirmed the superiority of metformin on cancer incidence or outcome, this would not change the current standard therapeutic approach for T2DM in the cancer patient. The debate about the association of specific antidiabetic drugs and cancer risk has been marred by the lack of properly designed studies.

Although observational studies suggest that the choice of treatment for DM may modify cancer risk [208], no prospective studies have been specifically designed to address this issue. Thus, no firm conclusions can be reached at this point. The crux of the debate has been whether insulin or analogs are associated to an increased risk of CRC (and cancer in general) [209] and whether metformin is associated with a decreased risk of CRC [210]. This may represent two sides of the same coin: if one drug does modify the risk of CRC, by comparison the other may appear to modify the risk in the opposite direction. Confounders may exist. Thus, insulin is generally prescribed and metformin remains formally contraindicated in advanced chronic kidney disease (CKD), a late event in the course of T2DM, despite recent clinical recommendations [211]. Renal insufficiency is associated with higher risk for all-cause cancer [212], although this association has not been demonstrated for CRC [213].

Recent meta-analyses have attempted to unravel the potential relationship between antidiabetic therapy and cancer or colorectal cancer. However, meta-analysis results heavily depend on the quality of the included studies. A recent meta-analysis involving approximately 7.6 million and 137, 540 patients with diabetes from observational studies and randomized controlled trials (RCTs), respectively, suggested that metformin or thiazolidinediones were associated with a lower risk of all cancer incidence, while insulin, sulfonylureas and alpha glucosidase inhibitors were associated with an increased risk of cancer incidence [214]. Another large (491, 384 individuals) meta-analysis addressing specifically the impact of insulin, found it to be associated with a significant 69% increased risk of CRC in T2DM only in case-control but not in cohort studies [215]. The Barcelona nested case-control study of 275, 164 T2DM patients did not find an increased risk of cancer for any insulin or oral antidiabetic agent [216]. Finally, a metaanalysis of 19 publications representing data for 1, 332, 120 individuals, insulin had no effect and insulin glargine was associated with a decreased risk of CRC [217].

Metformin use has been associated with a decreased risk of colon cancer and increased survival [210, 218, 219]. A systematic review of 12 randomized controlled trials (21, 595 patients) and 41 observational studies (1, 029, 389 patients) found that in observational studies the risk of CRC was 17% lower among DM patients treated with metformin than in those not on metformin [210]. In a meta-analysis of 21 observational studies metformin was associated with a reduction in cancer-specific mortality, including a reduction in mortality for colon cancer (4 studies, HR 0.65, 0.56-0.76) [205, 220]. Several mechanisms may account for the antitumor effect of metformin. It reduces circulating insulin, promotes weight loss and activates 5’ adenosine monophosphate-activated protein kinase (AMPK), thus inhibiting growth of colon cancer cells [221, 222]. In mice with Apc mutations, metformin suppressed polyp growth [223] and in diabetic mice metformin, alone or in combination with oxaliplatin, reduced the severity of colorectal tumors [224]. Older literature described increased expression of mitochondrial GPDH, the target of metformin, in rapidly growing, undifferentiated tumors [225, 226]. However, there are no data on CRC expression of mitochondrial GPDH. In non-diabetic subjects, oral short-term low-dose metformin suppressed the development of colorectal aberrant crypt foci in a clinical trial [227]. In phase 3 RCT, low-dose (250 mg/day) metformin was safe and reduced the prevalence and number of metachronous adenomas or polyps after polypectomy in non-diabetic patients [228].

Conflicting results are available on thiazolidinediones and cancer. A systematic review and meta-analysis of 840, 787 diabetic patients did not support an association between thiazolidinediones and CRC [229, 230]. In a 6-year population-based cohort study, thiazolidinediones were associated with decreased cancer risk including CRC and the association was dose-dependent [231]. Thiazolidinediones have cytostatic effects and inhibit growth and metastasis of colon cancer cells as they induce differentiation and modulate the E-cadherin/β-catenin system [232–234]. However, some studies point to a mitogenic potential of troglitazone which induced colon tumors in normal C57BL/6J mice and increased colon carcinogenesis in Apc1638 N/+Mlh1^+/−^ double mutant mice [235].

In systematic meta-analyses, sulphonylureas were associated with increased risk of pancreatic and hepatocellular cancer but not of CRC [229, 236, 237]. A cohort of 275, 164 T2DM patients found no evidence for altered cancer risk for repaglinide or α-glucosidase inhibitors compared to insulin-based therapies or other oral glucose-lowering drugs [216]. In other reports, acarbose was associated with reduced the risk of incident CRC in patients with diabetes in a dose-dependent manner [238, 239]. Acarbose may alter the microbiota [240] and decreased the size of gastrointestinal adenomas in Apc knockout mice [241].

Empaglifozin dramatically decreased mortality and slowed DKD progression and sodium-linked glucose transporter-2 (SGLT2) inhibitors may soon become the new standard of therapy [242]. A safety warning was issued by the FDA regarding bladder and breast cancer risk from early clinical trials of dapagliflozin but not for CRC (http://www.fda.gov/downloads/AdvisoryCommittees/CommitteesMeetingMaterials/Drugs/EndocrinologicandMetabolicDrugsAdvisoryCommittee/UCM262994.pdf). Adenocarcinomas express SGLT2 and SGLT2 inhibitors blocked glucose uptake and reduced growth of tumor xenografts [243]. Whether this applies to CRC is unknown.

No relationship between GLP-1-based therapies and CRC have been reported [244, 245]. However, exenatide inhibited proliferation and induced apoptosis in cultured murine CT26 colon cancer cells [246, 247].

### Choice of chemotherapy for colorectal rectal cancer in patients with diabetes

Studies addressing chemotherapy efficacy or safety in DM are very limited and there is no evidence supporting specific chemotherapy approaches for CRC patients with DM. No differences in the survival benefit or severe adverse effects associated to chemotherapy were observed in 5, 330 elderly CRC patients with (n=950) and without (n=4, 380) DM [248]. By contrast, a cohort study within the INT-0089 randomized adjuvant chemotherapy trial of 3, 759 patients with high-risk stage II/III colon cancer concluded that in DM patients overall mortality and cancer recurrence were higher than in non-diabetic patients [249]. Treatment-related toxicities were similar between DM and non-DM patients, except for a higher risk of treatment-related diarrhea among DM patients [249]. However, disease-free survival was lower and neurotoxicity more frequent in DM patients treated with capecitabine and oxaliplatin (CAPOX) chemotherapy than in non-diabetics [250]. It is likely that whether DM modifies the risk of severe adverse effects that limit chemotherapy depends on the specific chemotherapeutic regimen.

## AGENTS IN THE PIPELINE TARGETING BOTH CRC AND DIABETIC COMPLICATIONS

Some therapeutic targets are undergoing or have undergone RCTs in both diabetes complications (e.g. DKD) and cancer or are in clinical use in one condition and have been successfully used for the other condition in preclinical settings. These include statins, renin angiotensin aldosterone system (RAAS) blockers, endothelin receptor antagonists, VDR activators, mTOR inhibitors, anti-inflammatory molecules and inhibitors of EGF ligands/receptors (Table 4) [251, 252].

**Table 4:** Examples of agents in the pipeline targeting both cancer and diabetic target organ complications exemplified by diabetic kidney disease

Activity	Agent	Successful in animal models of cancer	Successful in experimental DKD	RCT in human cancer	RCT in human DKD	Refs
HMGCoA reductase inhibitors	statins	Yes	Yes	Yes	Yes	[253–256]
RAAS targeting drugs	ACE inhibitors, ARBs	Yes	Yes	No	Yes	[252, 257–259]
VDR activator	Paricalcitol	Yes	Yes	Yes	Yes	[265,266,291]
Endothelin receptor antagonists	Atrasentan and others	Yes	Yes	Yes	Yes	[262–264,292–301]
Anti-fibrotic agents	Anti-CTGF mAb FG3019	Yes	Yes	Yes	Yes	[101,302,303]
	Anti- TGF-β1 mAb.	Yes	Yes	Yes	Yes	[304]
Anti-inflammatory agents	Chemokine targeting agents	Yes	Yes	Yes (anti-CXCR4)	Yes (anti-CCL2 and others )	[270]
	JAK/STAT inhibitors	Yes	Yes	Yes	Yes	[276–279]
Inhibitors of epidermal growth factor Receptor/ligands	Several agents	Yes	Yes	Anti-EGFR antibodies (cetuximab)	Anti-TGF-α/epiregulin antibody (LY3016859)	[305,306]
mTOR inhibitors	Several agents	Yes	Yes	Yes	No (Yes in non-DKD CKD)	[98–99, 269]

DKD: diabetic kidney disease, CKD: chronic kidney disease, HMGCoA: 3-hydroxy-3-methylglutaryl coenzyme A, RAAS: renin angiotensin aldosterone system, ACE: angiotensin converting enzyme, ARB: angiotensin receptor blocker; CTGF: Connective tissue growth factor, TGF-beta: Transforming growth factor beta, EGFR: Epidermal growth factor receptor, CXCR4: Chemokine Receptor type 4, CCL2: Chemokine Ligand 2

Statins are commonly used to treat hyperlipidemia and have been linked with a small reduction in the risk for colon cancer in diabetic patients [253] and improved prognosis of curatively resected CRC [254]. In an obesity-related colon cancer model associated with hyperlipidemia and hyperinsulinemia, pitavastatin prevented carcinogenesis and inhibited colon proliferation and inflammation [255], while simvastatin inhibited the release of inflammatory cytokines by colorectal cell lines [256]. Clinical trials are exploring statins in the treatment of human CRC.

RAAS blockers are the mainstay of therapy for human DKD [252]. Angiotensin-converting enzyme inhibitors and angiotensin-II type 1 receptor blockers suppress chemically-induced colonic preneoplasic lesions in diabetic animals [257–259]. However, their clinical use to prevent colon cancer is not being pursued.

The endothelin receptor antagonist atrasentan is undergoing RCTs for DKD [260, 261], and as add-on to docetaxel and prednisone for stage IV hormone therapy-resistant prostate cancer bone metastases (NCT00134056) [262–264]. However, no trial is exploring CRC.

Paricalcitol is a VDR activator that may have antiproteinuric effects on DKD as suggested by RCTs [265] and may also slow cancer cell growth [266]. Phase I trials have tested combinations of paricalcitol and chemotherapeutic agents (NCT00217477). Additionally, vitamin D has been explored for colon cancer prevention. However, a combination of calcitriol, aspirin, and calcium carbonate or vitamin D/calcium did not prevent recurrence of colorectal adenomas over a 3- to 5-year period [267, 268].

mTOR inhibitors are used as anticancer agents and also improve experimental DKD [99, 269]. The mTOR inhibitors RAD001 (NCT01058655) and everolimus and the dual PI3K/mTOR inhibitor PF-05212384 (NCT01937715, NCT01154335) are undergoing clinical trials for metastatic CRC.

Several agents targeting cytokines and chemokines have been tested both in T2DM and cancer [251, 252, 270–273]. Plerixafor is a CXCR4 antagonist undergoing trials for advanced CRC (NCT02179970). Although not specifically tested in DM, a selective CXCR4 antagonist AMD3465 decreased mineralocorticoid-dependent renal fibrosis in mice [274] and targeting CXCR4 prevented glomerular injury associated to high podocyte CXCR4 expression in mice [275].

JAK2 targeting prevented high-glucose-induced fibrogenic responses in renal cells and prevented kidney and vascular injury in experimental diabetes [276–279]. An ongoing phase II RCT is testing the JAK1 and JAK2 inhibitor baricitinib as add-on to RAS blockade in patients with DKD (NCT01683409) while another will explore the JAK2/FLT3 inhibitor pacritinib in patients with refractory CRC and KRAS mutations (NCT02277093).

## UNANSWERED QUESTIONS AND THE WAY FORWARD

Table 5 summarizes the key points of the review. The association between DM and CRC is recognized by scientific consensus [4]. However, a number of issues require more detailed studies (Table 6).

**Table 5:** Key points

An epidemiological association has been reported between diabetes mellitus, especially type 2 diabetes mellitus , and colorectal cancer However, the association has evolved over time, there are differences between countries over the impact of sex, and colorectal cancer remains uncommon in many countries with a high prevalence of diabetes, suggesting the existence of poorly understood modifiers. The mechanistic basis for this association are poorly understood There are common risk factor for colorectal cancer, diabetes and diabetic complications There are controversial observational data on the association of antidiabetic drugs with colorectal cancer. The most convincing evidence is on a protective effect of metformin Preclinical data suggest that the diabetic environment may promote both colorectal cancer and diabetic complications. There is evidence derived from interventional preclinical studies, GWAS studies, and some interventional clinical data that suggest that CRC and well-characterized complications of diabetes, such as diabetic kidney disease, may share pathogenic pathways, including inflammatory mediators, an abnormal microbiota and altered iron metabolism, some of them converging at Wnt/β-catenin signaling and MIR-21. The finding of common pathogenic pathways for colorectal cancer and diabetic target organ complications (e.g. diabetic kidney disease) lend biological plausibility to the epidemiological observation However, to date no clinical practice consequence has derived from this knowledge

**Table 6:** Standing questions on the relationship between DM and colorectal cancer

Standing question	Relevance	What is required to address it
Is there an association between T2DM and colorectal cancer across all countries and cultures?	Provides insights into etiologic and pathophysiologic factors, may prevent a colorectal cancer epidemic in the developing world	Head-to-head comparison between developed and developing country cohorts
Is there an association between development of cancer and development of other complications of DM?	Provides the epidemiological basis to search for common mediators of disease	Epidemiological studies, ideally prospective
What molecular mediators explain the association between DM and cancer? Are they shared by other complications of DM?	Identification of potential diagnostic signatures and therapeutic targets	Interventional preclinical models that address function of key molecules. These may have been identified by non-biased systems biology approaches and hypothesis-driven studies designed from the analysis of available literature
Has DM-associated colorectal cancer a specific molecular signature?	This may identify diagnostic signatures and therapeutic targets specific for DM-associated colorectal cancer	Systems biology comparison between DM and non-DM associated colorectal cancer with DM and non-DM healthy colon as control
Can DM patients at high risk for cancer development be identified by diagnostic tests?	Early diagnosis of risk or cancer	Prospective systems biology approach to relevant biological samples (feces, urine, blood or others)
Can DM patients at high risk or early colorectal cancer be treated by specific, DM-tailored approaches? Do these approaches also prevent/treat other diabetic complications?	New preventive/therapeutic approaches that address both cancer and non-cancer DM complications	Early identification of patients at high risk or with early disease Unraveling of common pathogenic pathways
Are there common microbiota signatures for colorectal cancer and other DM complications?	New preventive/therapeutic approaches that address both cancer and non-cancer DM complications	Metagenomic studies
What is the optimal therapeutic approach for colorectal cancer in diabetic individuals and the optimal therapeutic approaches for DM in colorectal cancer patients?	Therapy individualization and improved outcomes	Hypothesis-generating observational studies followed by randomized clinical trials

An overview of T2DM and CRC country-based prevalence/incidence suggests that environmental, development-associated or other factors may interact with the T2DM milieu to increase the risk of CRC. Identification of these putative factors and whether DM associates with increased CRC risk in different cultures and countries may provide further insights into mechanisms underlying the relationship between DM and cancer.

The case for a causal association should be strengthened by the characterization of the DM-initiated molecular pathways involved. This information may also lead to the development of specific preventive or therapeutic approaches. Studies should address the relationship between DM-associated CRC and the development of other DM-associated complications, i.e., whether there is a patient profile prone to develop any DM-related complications. If this were the case, tools should be developed for the early identification of such patients. Urine proteomics holds promise in this regard, as it allows identification of DKD at earlier stages than currently available methods and predicts progression [280, 281] and may also be useful for the diagnosis of cancer outside the urogenital system [282]. Early identification of the subpopulation of DM patients at highest risk for developing cancer or classical complications may allow enrollment in trials assessing the efficacy of drugs targeting shared molecular mechanisms for prevention and/or therapy. Additional systems biology approaches may also contribute to define molecular pathways leading to DM-associated cancer or target organ damage. The most promising approaches should undergo clinical trial testing, ideally in high-risk populations or in early disease stages identified by the study of specific molecular signatures.

Research is needed to define the optimal therapeutic approach for the patient with T2DM and CRC. Studies of the impact of different antidiabetic agents on cancer incidence are marred by the fact that both sides of the comparison may theoretically modulate cancer incidence. Additionally, there are potential biases related to the indication of the specific agent. These research efforts have the potential to decrease the incidence of DM-associated complications and to improve outcomes. The DiabetesCancerConnect Consortium funded by the Spanish Government is attempting to answer some of these questions.

## SUPPLEMENTARY MATERIALS FIGURES AND TABLES



## Abbreviations

DM: Diabetes mellitus
CRC: Colorectal cancer
ADA: American Diabetes Association
T1DM: Type 1 DM
T2DM: Type 2 DM
MODY: Maturity-onset diabetes of the young
GLP-1: Glucagon like peptide-1
GPDH: Glycerophosphate dehydrogenase
GPD2: Glycerol-3-phosphate dehydrogenase 2
GSK-3β: Glycogen synthase kinase 3-beta
EGFR: Epidermal growth factor receptor
HR: Hazard Ratio
SIR: Standardized incidence ratios
RR: Relative risk
CI: Confidence interval
BMI: Body mass index
OR: Odds Ratio
HbA1c: Glycated hemoglobin
AGEs: Advanced Glycation End-products
RELMβ: Resistin-like molecule β
IGF-1: Insulin-like growth factor
MAPK: Mitogen activated protein kinase
PI3K: Phosphatidyl-inositol-3-kinase
PAK-1: Activated protein kinase-1
mRNA: Messenger RNA
ChREBP: Carbohydrate response element-binding protein
NF-κB: Factor-kappa B
Nrf2: Nuclear factor erythroid 2–related factor 2
ATP: Adenosine triphosphate
Glut-1: Glucotransporter-1
TGF: Transforming growth factor
VDR: Vitamin D receptor
PTHrP: Parathyroid hormone-related protein-
Grem1: Gremlin
S1P: Sphingosine-1-phosphate
iHMP: Integrative Human Microbiome Project
TACT: Trial to Assess Chelation Therapy
EDTA: Ethylenediaminetetraacetic acid
miRNAs: MicroRNAs
GWAS: Genome-wide association studies
lncRNA: Long non-coding RNA
SNP: Single Nucleotide Polymorphism
eQTL: Expression quantitative trait loci
KEGG: Kyoto Encyclopedia of Genes and Genomes
VEGFR: Vascular endothelial growth factor receptor
B/D-HPP: Biology/Disease-driven Human Proteome Project
HDPP: Human Diabetes Proteome Project
SELENBP1: Selenium-binding protein 1
CAI: Carbonic anhydrase I
DSPathNet: Drug-specific Signaling Pathway Network
RCTs: Randomized controlled trials
CKD: Chronic kidney disease
ROS: Reactive Oxygen species
PPARγ: peroxisome proliferator–activated receptor γ
DNA: Deoxyribonucleic acid
RNA: Ribonucleic acid
FDA: Food and Drug Administration
CAPOX: Capecitabine and oxaliplatin
RAAS: Renin angiotensin aldosterone system
mAb: Monoclonal antibody
HG: Hyperglycemia
DKD: Diabetic kidney disease
ARB: Angiotensin receptor blocker
HMGCoA: 3-hydroxy-3-methylglutaryl coenzyme A
ACE: Angiotensin converting enzyme-
iNOS: Inducible nitric oxide synthase
NF-κB: Nuclear factor kappa-light-chain-enhancer of activated B cells
ERK: Extracellular Signal-regulated Kinase
CTGF: Connective tissue growth factor
TGF-beta: Transforming growth factor beta
CXCR4: Chemokine Receptor type 4
CCL2: Chemokine Ligand 2
