# Supplementary material for: 2017 update on the relationship between diabetes and colorectal cancer: epidemiology, potential molecular mechanisms and therapeutic implications
Source: Oncotarget. 2017 Jan 3;8(11):18456–85. doi: 10.18632/oncotarget.14472 (PMC5392343; doi:10.18632/oncotarget.14472)
Supplement: Supplementary file 1 [file oncotarget-08-18456-s001.pdf]

2017 update on the relationship between diabetes and colorectal cancer: epidemiology, potential molecular mechanisms and therapeutic implications

Supplementary Material

Supplementary Table 1: Agents to treat DM

	Agent	Route	Molecular target	Impact on glucose homeostasis and insulin	Impact on weight	Impact on colorectal cancer
	**Insulin**	Parenteral	Activates insulin receptor	Increased insulin	Increase	Increase/neutral/ decrease
**Activate insulin receptor/ increase insulin secretion**	**Sulphonyl-ureas/ Megli-tinides**	Oral	Inhibits sulfonylurea receptor (SUR1, a component of the ATP-dependent potassium channel)/ modulate ATP-dependent potassium channel activity in pancreatic beta cells	Increased insulin secretion	Increase	Neutral
	GLP-1-based therapies: **DPP-4 inhibitors**	Oral	Inhibits **DPP-4 and decreases degradation of incretins**	Enhanced glucose-dependent insulin secretion, slowed gastric emptying reduced postprandial glucagon and food intake	Neutral	Neutral
	GLP-1-based therapies: GLP-1 receptor agonists	Parenteral	Activates GLP-1 receptor		Neutral/ decrease	Neutral/decrease
**Sensitize to insulin action**	**Thiazolidin-ediones**	Oral	Activate peroxisome proliferator-activated receptor gamma (PPAR-γ)	Sensitize to insulin action: increase glucose utilization and decrease glucose production	Neutral	Neutral/decrease
**Decrease glucose absorption or synthesis**	**Metformin**	Oral	Inhibits glycerophosphate dehydrogenase	Decrease glucose synthesis	Decrease	Decrease
	**α-glucosidase inhibitors**	Oral	Inhibit intestinal **alpha-glucosidase**	Decrease glucose gut absorption	Decrease	Neutral/decrease
	SGLT2 inhibitors	Oral	Inhibit sodium-linked glucose transporter 2	Increase glucose urinary losses	Decrease	Neutral

GLP-1: Glucagon-like peptide-1; DPP-4: dipeptidyl peptidase 4; SGLT2: sodium-linked glucose transporter
GLP-1-based therapies mimic the effects of incretins. Incretins (GLP-1 and glucose-dependent insulinotropic polypeptide) are gut hormones released in response to food ingestion that stimulate insulin secretion and limit glucagon release.
